# Roles of the HUWE1 ubiquitin ligase in nervous system development, function and disease

**DOI:** 10.1186/s13064-020-00143-9

**Published:** 2020-04-26

**Authors:** Andrew C. Giles, Brock Grill

**Affiliations:** Department of Neuroscience, The Scripps Research Institute, Jupiter, Florida, 33458 USA

**Keywords:** Huwe1, EEL-1, Ubiquitin ligase, HECT, Neuron, Neural progenitor, Neurotransmission, Axon, Transcription factor, Intellectual disability

## Abstract

Huwe1 is a highly conserved member of the HECT E3 ubiquitin ligase family. Here, we explore the growing importance of Huwe1 in nervous system development, function and disease. We discuss extensive progress made in deciphering how Huwe1 regulates neural progenitor proliferation and differentiation, cell migration, and axon development. We highlight recent evidence indicating that Huwe1 regulates inhibitory neurotransmission. In covering these topics, we focus on findings made using both vertebrate and invertebrate in vivo model systems. Finally, we discuss extensive human genetic studies that strongly implicate HUWE1 in intellectual disability, and heighten the importance of continuing to unravel how Huwe1 affects the nervous system.

## Introduction

**Fig. 1:**
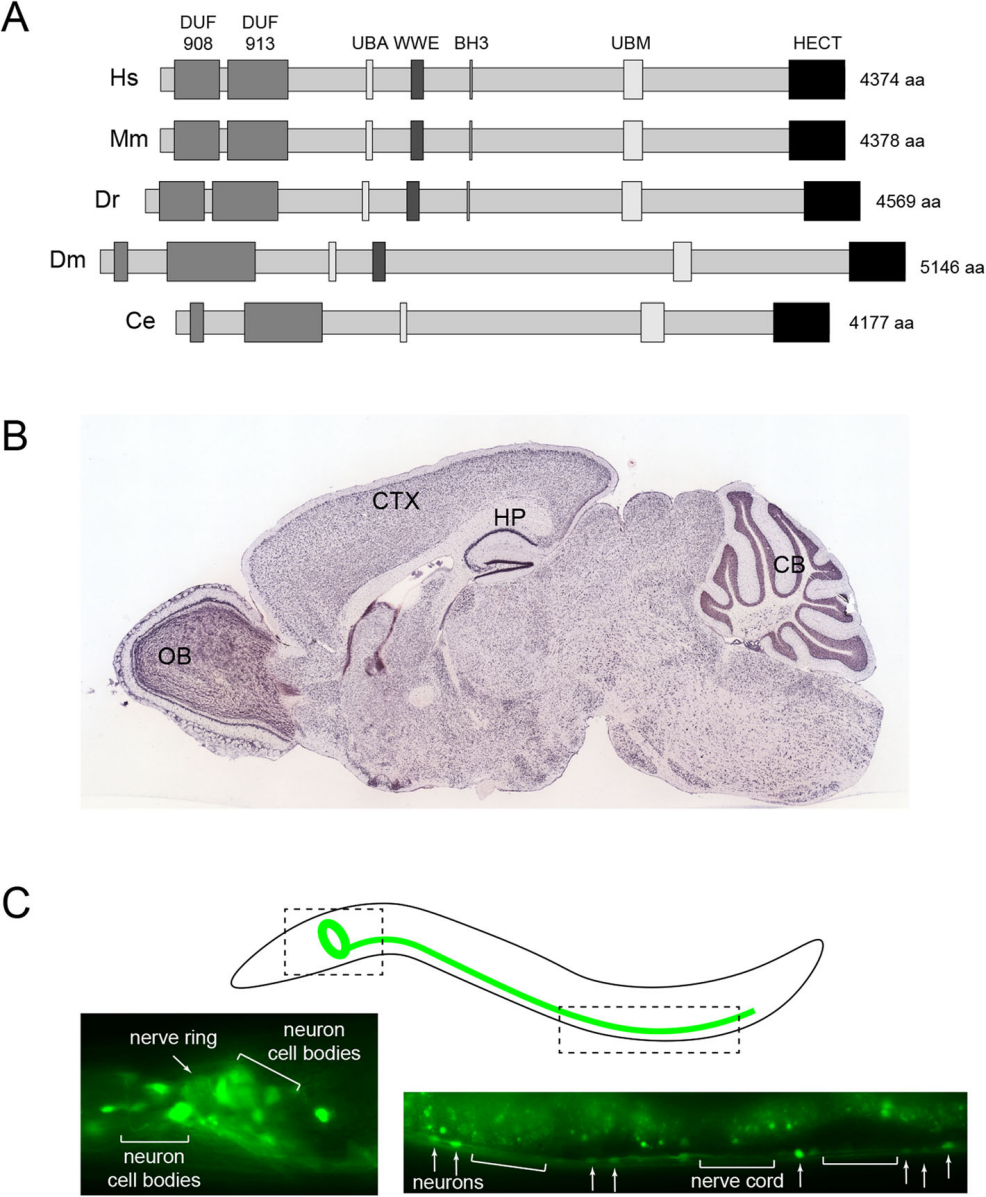
HUWE1 is evolutionarily conserved and broadly expressed in animal nervous systems. **a** Domain architecture of Huwe1 orthologs from *Homo sapiens* (Hs), *Mus musculus* (Mm), *Danio rerio* (Dr), *Drosophila melanogaster* (Dm) and *Caenorhabditis elegans* (Ce). **b** In situ hybridization of *Huwe1* in mouse brain. Note *Huwe1* is expressed throughout the brain with high expression in olfactory bulb (OB), cerebral cortex (CTX), hippocampus (HP) and cerebellum (CB). **c***eel-1* promoter driving GFP expression in *C. elegans*. *eel-1* is widely expressed in the nerve ring and head neurons (left), and ventral nerve cord (right; bars = nerve cord; arrows = motor neuron cell bodies). Schematic denotes locations of images shown and axon bundles of the nerve ring and ventral nerve cord. DUF = conserved Domain of Unknown Function, UBA = Ubiquitin Associated domain, WWE = domain with conserved tryptophan (W) and glutamate (E) residues, UBM = Ubiquitin-Binding Motif (previously known as DUF4414), HECT = Homologous to the E6-AP Carboxyl Terminus (HECT)-type E3 ubiquitin ligase domain. For B, image credit: Allen Institute© 2007 Allen Institute for Brain Science. Allen Mouse Brain Atlas. Available from: https://mouse.brain-map.org/experiment/show/70445293. For C, images reprinted from Cell Reports, Vol. 19, Opperman, Mulcahy, Giles, Risley, Birnbaum, Tulgren, Dawson-Scully, Zhen, Grill, The HECT Family Ubiquitin Ligase EEL-1 Regulates Neuronal Function and Development, 822–835, Copyright 2017, with permission from Elsevier

HECT, UBA and WWE domain containing protein 1 (Huwe1) is an E3 ubiquitin ligase that is highly conserved across the animal kingdom [1–4] (Fig. 1a). While rodent, zebrafish and fly orthologs are also generally referred to as Huwe1, the *C. elegans* ortholog is called Enhancer of EFL-1 (EEL-1) based on its discovery in genetic screens. Early studies found that Huwe1 was involved in regulating tumor cell proliferation/suppression, apoptosis, responses to DNA damage and embryogenesis [2–8]. Many of these initial studies refer to Huwe1 by other names including Mule, ArfBP1, Lasu1, HectH9 or Ureb1. More recently, Huwe1 was shown to have important roles in the immune system and myogenesis [9–11].

A growing body of work over the last 10 years, spanning multiple model systems, has shown that Huwe1 is important in nervous system development and function. Extensive human genetic studies have provided compelling evidence implicating Huwe1 in multiple neurodevelopmental disorders, including both non-syndromic and syndromic forms of X-linked intellectual disability (ID). In this review, we discuss how Huwe1 contributes to nervous system development, function and disease. We hope that exploring this literature encourages further studies on Huwe1 in the nervous system, and highlights valuable directions for future investigation from both a basic science and disease perspective.

## Huwe1 protein composition and nervous system expression

Huwe1 is a ubiquitin ligase in the HECT family, a group of E3 ubiquitin ligases that function as single subunit enzymes [5, 12]. A striking feature of Huwe1 is its enormous size — well over 4000 amino acids (Fig. 1a). Huwe1 has numerous protein domains including a catalytic ubiquitin ligase domain, several domains involved in ubiquitin binding, and multiple conserved domains of unknown function (Fig. 1a and Fig. 4). The best characterized domain is the HECT domain, which is located at the C-terminus and is the catalytic E3 ubiquitin ligase domain that conjugates ubiquitin to substrates [13, 14]. Other domains involved in ubiquitination include a UBA domain that binds polyubiquitin chains, a WWE domain that mediates protein-protein interactions during ubiquitination, and a ubiquitin binding motif (UBM) that contains several repeated sequences and binds ubiquitin (previously called DUF4414). There is also an extremely small Bcl-2 homology domain 3 (BH3) that is a protein-protein interaction region, and only present in vertebrate isoforms of Huwe1. Two studies have solved the structure of the HECT domain [13, 14]. Interestingly, the Huwe1 HECT domain forms a dimer, which leads to autoinhibition of catalytic ubiquitin ligase activity [14]. At present, the functional roles of many domains in Huwe1 remain unknown, and the biochemical mechanisms that allow for substrate specificity are unclear. The full complement of proteins that interact with Huwe1 and potential non-ubiquitin ligase mechanisms of Huwe1 function also remain largely unexplored.

Interest in Huwe1 function in the nervous system becomes apparent when one simply considers how broadly expressed this molecule is across animal nervous systems. In both humans and rodents, Huwe1 is expressed throughout the adult brain with particularly strong expression in the olfactory bulb, superficial layers of the cortex, hippocampus and cerebellum (Fig. 1b) [15–17]. In adult *C. elegans*, the Huwe1 ortholog EEL-1 is expressed broadly throughout the nervous system. This includes sensory neurons, the nerve ring (a central bundle of hundreds of axons), and the motor neurons of the nerve cord (Fig. 1c) [18]. The broad expression of Huwe1 in adult mammalian and *C. elegans* nervous systems is consistent with Huwe1 having widespread, important roles in the nervous system, which we discuss further.

## Huwe1 ubiquitin ligase activity in neural progenitor proliferation and differentiation

**Fig. 2:**
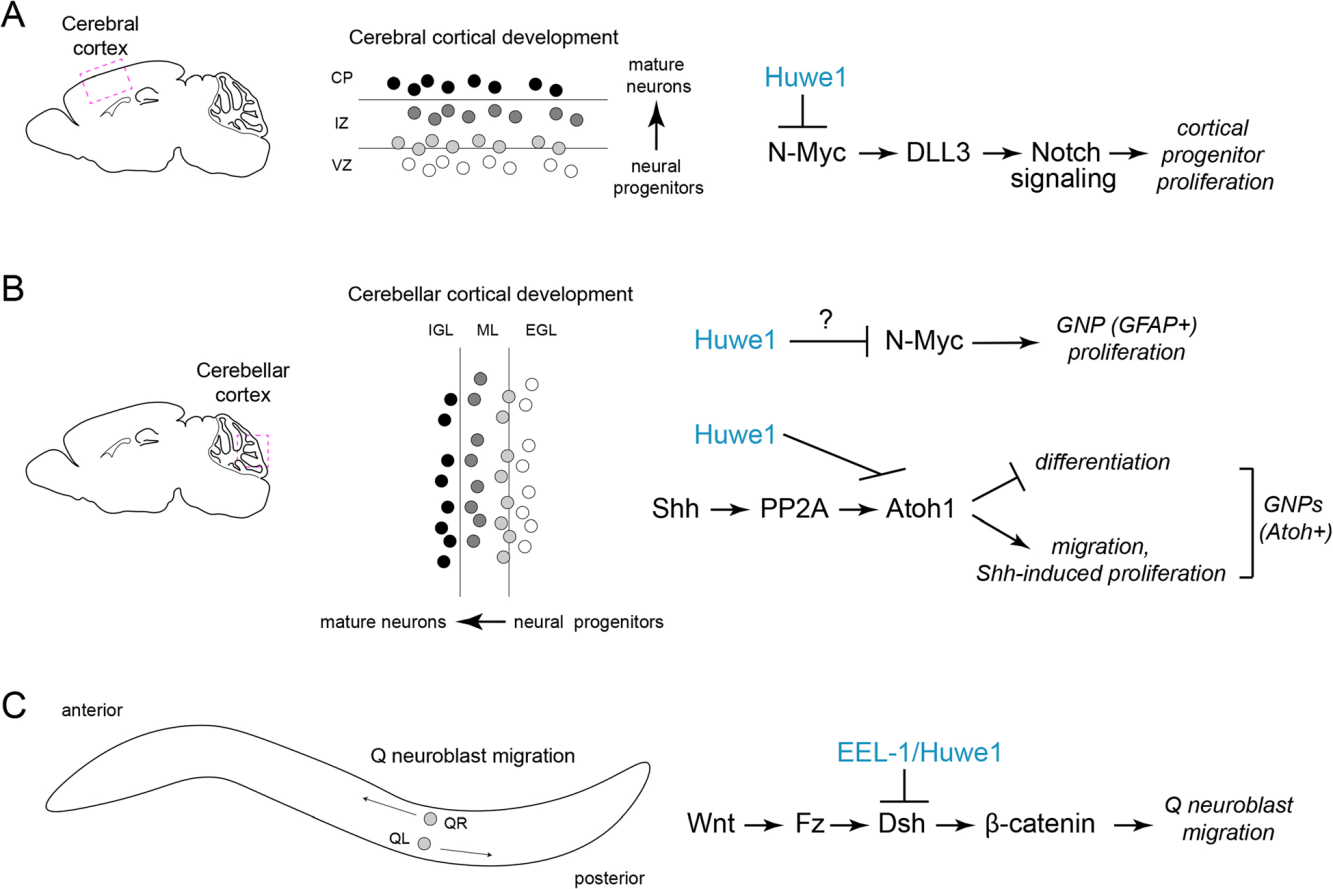
HUWE1 regulates neural development. (**a** and **b**) Shown to the left are diagrams of sagittal section of mouse brain. Boxes indicate a portion of (**a**) cerebral cortex and (**b**) cerebellar cortex. Center is a schematic of Huwe1 expression in neural progenitors that are proliferating, differentiating and migrating during development in (**a**) cerebral cortex and (**b**) cerebellar cortex (white = low expression, black = high expression). To the right are molecular pathways Huwe1 regulates to affect progenitor development in (**a**) cerebral cortex and (**b**) cerebellar cortex. Note that in the cerebellum Huwe1 potentially affects two pathways in different GNP populations. **c** Shown is a schematic of Q neuroblast migration in *C. elegans* which is mediated by Wnt signaling. To the left is the molecular mechanism by which EEL-1 and HUWE1 influence Wnt signaling in neuroblast migration and mammalian cell-based assays

Mammalian nervous systems are composed of millions to billions of neurons. Waves of cell proliferation in various regions of the developing brain are critical to achieve these large numbers. Two particularly important regions for brain growth are the ventricular zone (VZ) of the neocortex (Fig. 2a) and the external granule layer (EGL) of the cerebellum (Fig. 2b). Proliferation of neural stem cells and neural progenitors takes place in deep layers of the neocortex. As precursors differentiate into neurons they migrate to the cortical plate (CP). In the cerebellum, proliferation of neural progenitors occurs in the superficial EGL and differentiating neurons migrate deeper to the internal granule layer (IGL). Interestingly, Huwe1 expression is lowest in regions where proliferation of neural stems cells and progenitors occurs and expression gradually increases away from these regions where neurons are differentiating and migrating [15]. Consistent with these expression patterns, both cell-based and in vivo approaches have shown that Huwe1 plays prominent roles in early development of the nervous system.

The first cell-based experiments focused on differentiation of cultured embryonic stem (ES) cells into a neuronal lineage [15]. Both germline and conditional knock out of *Huwe1* impaired ES cell differentiation. In cultured mouse brain slice, *Huwe1* knockdown and knockout increased proliferation of cortical progenitors and reduced differentiation to neurons [15]. A subsequent in vivo study conditionally deleted the Huwe1 ubiquitin ligase domain broadly in neurons [19]. *Huwe1* conditional knockouts (cKO) displayed neonatal lethality within 24 h of birth. Increased numbers of neural progenitors were observed in the developing cortex of *Huwe1* cKO animals further solidifying the concept that Huwe1 inhibits neural progenitor proliferation. Consistent with this, overexpression of Huwe1 in cultured cortical slices caused the opposite phenotype, fewer dividing cells. Huwe1 ubiquitin ligase activity also functions later in development, as Huwe1 ubiquitin ligase cKOs displayed defects in differentiation of neurons in the cortex [19]. Taken together, these studies indicate that Huwe1 ubiquitin ligase activity inhibits proliferation of neural progenitors early in development, and encourages neuronal differentiation during cortical development. These functions of Huwe1 are required for proper patterning of the cortex.

Because *Huwe1* cKO in neurons throughout the brain resulted in lethality [19], it was plausible Huwe1 had potential roles in nervous system development outside the cortex. This was tested when Lasorella and colleagues conditionally deleted Huwe1 ubiquitin ligase activity in granule neuron progenitors (GNPs) and radial glia of the cerebellum using the *GFAP* promoter to express Cre [20]. 50% of these *Huwe1* cKO mice died within 4 weeks of birth. Survivors developed ataxia consistent with cerebellar dysfunction. Similar to results in cortex, cerebellar GNPs lacking Huwe1 displayed expanded proliferation resulting in increased GNP numbers, increased EGL size, and reduced numbers of mature neurons in the IGL [20]. Abnormalities in cerebellar glia were also observed.

GNPs of the developing cerebellum are not a homogenous population of cells. As a result, when Huwe1 was conditionally knocked out in a subset of GNPs that expressed the Atoh1 transcription factor, GNP differentiation was impaired [21]. Proliferation of Atoh1-positive GNPs could be affected in *Huwe1* cKOs, but only when proliferation was increased in culture using the mitogenic stimulant Sonic Hedge Hog (Shh). Thus, compelling evidence from cell culture, brain slice, and multiple intact developing brain regions indicates that Huwe1 ubiquitin ligase activity is critical in regulating the switch from proliferation to differentiation in neural progenitors.

## Huwe1 affects multiple transcriptional programs to influence proliferation and differentiation

Let us now comment on the mechanisms by which Huwe1 regulates neural progenitor proliferation and differentiation early in development. As noted above, knockout strategies in which the HECT ubiquitin ligase domain was conditionally deleted indicated that Huwe1 ubiquitin ligase activity is required to switch neural progenitors from a proliferative state towards differentiation in the developing nervous system. Genetic and biochemical results indicated that this occurs because Huwe1 can ubiquitinate and inhibit two transcription factors, N-Myc and Atoh1 [15, 19, 21]. Consistent with this, N-Myc and Atoh1 are expressed in proliferating neural stem cells and progenitors, are down regulated as cells differentiate and can affect neural progenitor proliferation and/or differentiation.

Affinity purification proteomics with a human neuroblastoma cell line initially identified Huwe1 as an N-Myc binding protein [15]. Several further in vitro and cell-based biochemical experiments indicated that Huwe1 polyubiquitinates N-Myc resulting in degradation by the 26S proteasome. Experiments in ES cells, cultured brain slice and intact brain showed that Huwe1 inhibits N-Myc to affect proliferation and differentiation of neural stem cells and progenitors. For example, Huwe1 knockout in ES cells impairs differentiation towards neural cell fates, which is rescued by N-Myc knockdown [15]. Consistent with this, levels of the N-Myc transcriptional target Cyclin D2 are elevated when Huwe1 is impaired. Similar results in cultured brain slice indicate that increased proliferation and decreased differentiation of neural progenitors caused by *Huwe1* knockdown is rescued by *N-Myc* knockdown [15]. In developing mouse brain, *Huwe1* cKO in cortex resulted in increased neuronal progenitor proliferation that was suppressed by N-Myc knockdown [19]. In both the cortex and cerebellum, loss of Huwe1 function increased N-Myc expression, and altered the N-Myc transcriptional target Cyclin D2 [19, 20]. These results indicate that Huwe1 inhibits N-Myc to affect cortical progenitor proliferation. Effects of Huwe1 on N-Myc might also influence cerebellar progenitor proliferation, but this remains unclear.

Another principle gene affected by Huwe1 inhibition of N-Myc is the Notch ligand DLL3 [19]. DLL3 was discovered as a possible downstream effector of N-Myc by transcriptomics with a large number of gliomas that had differing levels of Huwe1 expression. *DLL3* was confirmed as a transcriptional target of N-Myc, as N-Myc binds the *DDL3* promoter and ectopic N-Myc expression increases DLL3 levels and Notch signaling. Consistent with this, Huwe1 degradation of N-Myc influences DLL3 [19]. For example, *Huwe1* cKO animals display increased *DLL3* mRNA and Notch1 signaling in the brain. In cultured brain slice, *Huwe1* cKO causes increased neural progenitor proliferation which is suppressed by knocking down *DLL3* or *N-Myc*. Collectively, this is strong evidence that Huwe1 directly inhibits N-Myc thereby affecting *DLL3* transcription and, ultimately, neural progenitor proliferation and differentiation in the developing cortex (Fig. 2a).

In the cerebellum, Huwe1 inhibition of N-Myc might affect proliferation of GNPs that express N-Myc [20], but this remains untested. A subset of cerebellar GNPs express the Atoh1 transcription factor. Huwe1 ubiquitinates and degrades Atoh1, and robustly affects development of GNPs that express this transcription factor [21]. This discovery initially stemmed from affinity purification proteomics using HEK 293 cells, which identified Huwe1 as an Atoh1 binding protein. Cell-based experiments showed that Huwe1 knockdown and overexpression affect Atoh1 ubiquitination and stability. Further supporting these findings, *Huwe1* cKO resulted in increased Atoh1 protein levels in GNPs in culture and in vivo.

Overexpression of Atoh1 in cultured, purified GNPs impairs differentiation, but does not affect progenitor proliferation [22]. Consistent with Huwe1 being a negative regulator of Atoh1, *Huwe1* cKOs where the *Atoh1* promoter was used to drive Cre expression mimicked Atoh1 overexpression phenotypes with decreased differentiation of cultured GNPs, but no effect on GNP proliferation [21]. In cultured cerebellar brain slice, *Huwe1* knockdown results in impaired differentiation of GNPs. This effect was strongly suppressed when *Huwe1* and *Atoh1* were simultaneously knocked down [21]. Further experiments evaluating GNP differentiation in vivo using conditional knockout alleles for both *Huwe1* and *Atoh1* will be valuable in further evaluating this genetic relationship. Nonetheless, existing results with knockdown approaches taken together with extensive, high-quality biochemical studies support the conclusion that Huwe1 ubiquitinates and inhibits Atoh1 to affect differentiation of a subset of GNPs in the cerebellum.

Interestingly, Huwe1 can inhibit proliferation of GNPs expressing Atoh1, but this only occurs when GNPs are deprived of and re-treated with Shh to stimulate proliferation [21]. This likely occurs because Shh signaling through PP2A affects dephosphorylation of Atoh1 at two serine residues. Atoh1 dephosphorylation then prevents Huwe1 ubiquitination and degradation. Thus, Huwe1 and Shh-PP2A signaling display differing effects on Atoh1 stability, which influences proliferation of Atoh1 expressing GNPs (Fig. 2b).

Work on cochlear development also found that Huwe1 ubiquitinates and inhibits Atoh1 to regulate hair cell differentiation [23]. This independent study reproduced proteomic and biochemical findings to confirm that Huwe1 binds and ubiquitinates Atoh1. Interestingly, Casein Kinase 1 (CK1) phosphorylation of Atoh1 affected ubiquitination by Huwe1. This provides further evidence that phosphorylation of Atoh1 affects Huwe1-mediated degradation, and demonstrates that Huwe1 inhibits Atoh1 to affect cellular differentiation in multiple cell types.

In summary, Huwe1 restricts genetic programs required to switch from proliferation to differentiation in multiple types of neural progenitors. This occurs by Huwe1 ubiquitin ligase activity triggering proteasomal degradation and inhibition of the N-Myc and Atoh1 transcription factors. This compelling body of work on Huwe1 in early nervous system development has several future research directions poised to be particularly fruitful. 1) Determining how Huwe1 is regulated in the nervous system. 2) Identifying Huwe1 binding proteins that are not ubiquitination substrates, but potentially function with this enormous molecule. 3) Evaluating if further Huwe1 ubiquitination substrates, that are novel or previously identified in cell-based studies, have functional relevance in early nervous system development.

There is one final point regarding nervous system disease that is worth making here. Genetic programs involved in early development of the nervous system are often hijacked in brain cancers. Indeed, genetic changes in *HUWE1* are implicated in patients with glioblastoma and medulloblastoma [19, 21]. Whether HUWE1 is involved in neuroblastoma remains unknown. These intriguing initial links to cancer now encourage further research aimed at exploring how broadly and frequently *HUWE1* mutations occur in different brain cancers.

## Huwe1 and adult stem cell proliferation

Huwe1 can also affect neural stem cell proliferation after completion of the developmental program. In the adult hippocampus, neural stem cells in the dentate gyrus must maintain a balance between proliferation and quiescence. Too much proliferation and the stem cell compartment accumulates DNA damage and becomes exhausted; an excess of quiescence results in a dearth of new neurons. When Huwe1 was conditionally knocked out in adult animals using tamoxifen-inducible Cre, stem cell proliferation increased and differentiation decreased [24]. Experiments that varied the timing of cell cycle labelling relative to the induction of *Huwe1* cKO revealed that Huwe1 has a prominent role in promoting quiescence of stem cells that are in a proliferative state. Experiments with *Huwe1* cKO after 5 months indicated that defects in neural stem cell quiescence caused by loss of Huwe1 function result in long-term defects in proliferation. Thus, Huwe1 is needed to restrain proliferation, ensure a return to quiescence, and promote long-term maintenance of the adult neural stem cell compartment.

Huwe1 affects adult stem cell proliferation by ubiquitinating and degrading the transcription factor Ascl1 [24]. AscI was initially identified as a Huwe1 ubiquitination substrate using affinity purification proteomics from purified hippocampal stem cells. Ascl1 polyubiquitination was inhibited by *Huwe1* knockdown and Ascl1 stability was increased when *Huwe1* was conditionally knocked out in cultured neural stem cells. Huwe1 polyubiquitination of Ascl in neural stem cells was independently verified [25]. Genetic outcomes support these biochemical and proteomic findings, as *Huwe1* cKO in adulthood resulted in increased Ascl1 expression and increased numbers of Ascl1 expressing neural progenitors in the hippocampus [24]. This was accompanied by reduced differentiation of stem cells into neural progenitors. Consistent with Huwe1 inhibiting Ascl1, the Ascl1 transcriptional targets *Cyclin D1* and *D2* were elevated in stem cells lacking Huwe1.

These results suggest that Huwe1 constrains Ascl1 transcription factor levels in neural stem cells to restrict proliferation and promote quiescence in the hippocampus of adult mice. Going forward, genetic experiments in which *Huwe1* and *Ascl1* are both eliminated will be important to solidify this intriguing discovery.

## Huwe1 is a conserved regulator of neuronal migration

It can be challenging to dissect how genetic perturbations affect differentiation and migration of maturing neurons because problems with differentiation can indirectly influence migration. Nonetheless, because genetic manipulation of *Huwe1* caused consistent, conserved effects on migration, this warrants commentary. Furthermore, studies using *C. elegans* have provided valuable insight into the molecular mechanisms Huwe1 influences to specifically affect neuroblast migration.

Huwe1 effects on neural migration have been most extensively studied by evaluating granule neuron migration in the cerebellum [20]. Normally, large numbers of differentiating granule neurons migrate from the EGL through the molecular layer (ML) to the IGL (Fig. 2b). In *Huwe1* cKOs, few cells reach the IGL, and most cells are trapped in the ML or EGL layers. It is important to note that *Huwe1* cKO in cerebellum also affects the organization of Bergmann glial cells [20]. Bergmann glia provide a structural scaffold and cues for migrating granule neurons in the developing cerebellum. Thus, granule neuron migration defects in *Huwe1* cKOs are potentially caused by a combination of differentiation defects in GNPs and abnormalities in Bergmann glia. Experiments with cultured brain slice delved into the molecular mechanism by which Huwe1 affects granule neuron migration in the cerebellum [21]. *Huwe1* knockdown in developing cerebellum dramatically impaired migration of GNPs from the EGL to the IGL. These defects were mimicked by overexpression of Atoh1, a transcription factor and Huwe1 ubiquitination substrate. Importantly, double knockdown of *Huwe1* and *Atoh1* suppressed migration defects caused by *Huwe1* single knockdown. These results indicate that Huwe1 inhibits Atoh1 to promote GNP migration in cerebellum (Fig. 2b).

*C. elegans* has also yielded important insight into how Huwe1 regulates neuroblast migration during early nervous system development. As noted earlier, *C. elegans* has a single, highly conserved Huwe1 ortholog called EEL-1. In vivo genetic results indicate EEL-1 has a pronounced role in Wnt-mediated migration of Q neuroblasts [26]. Because Wnt signaling regulates Q neuroblast migration, when Wnt secretion is reduced in *vps-29* mutants the left Q neuroblast (QL) progeny migrate incorrectly to the anterior. This genetic background was used to identify regulators of Wnt signaling. An RNAi screen against a swath of ubiquitin machinery found that *eel-1* knockdown suppressed migration defects in *vsp-29* mutants. Cell-specific knockdown confirmed that EEL-1 functions cell autonomously in Q neuroblasts. These results indicated that EEL-1 likely functions downstream of Wnt signaling. Further genetic results showed that EEL-1 functions upstream of the β-catenin BAR-1, a Wnt signaling effector. Biochemical experiments using HEK 293 cells demonstrated that Huwe1 ubiquitinates Dishevelled, and Wnt stimulates this ubiquitination [26]. Proteomic and coimmunoprecipitation results indicated endogenous Huwe1 binds two Dishevelled isoforms (Dvl2 and Dvl3) in cultured murine embryonic fibroblasts. Similar to findings on Huwe1 ubiquitination of Atoh1 during cochlear development [23], CK1 affects Huwe1 ubiquitination of Dishevelled [26]. When *Huwe1* was knocked down and evaluated in cell-based assays for Wnt signaling, outcomes indicated that Huwe1 can act as a conserved inhibitor of Wnt signaling. Interestingly, Huwe1 ubiquitination does not trigger proteasomal degradation of Dishevelled, but rather blocks its activation thereby preventing downstream signaling to β-catenin. These findings demonstrate that Huwe1 is a conserved inhibitor of Wnt signaling that influences neuroblast migration in *C. elegans* in vivo (Fig. 2c).

Going forward, it will be important to know if Huwe1 affects Wnt signaling to influence neural migration in other organisms. Since Wnts affect axon polarity, axon guidance, synapse formation and synaptic plasticity [27], it could be valuable to explore whether Huwe1 plays a role in these events. This is encouraged by findings from Drosophila (discussed below) that show Huwe1 can affect axon development [28], a possible interaction between Huwe1 and another ubiquitin ligase that affects Wnt signaling [29], and evidence that Huwe1 affects Wnt signaling during intestinal oncogenesis [30]. Studies spanning rodent and *C. elegans* model systems also bring to the forefront several questions about Huwe1 and cell migration. Can it be more definitively determined if Huwe1 effects on migration are a secondary consequence of altered progenitor differentiation, the result of Huwe1 function in glia, or a combination of both? How widespread a role does Huwe1 have in neural migration? Finally, can future experiments provide evidence that Huwe1 directly effects cell migration signaling in mammals in vivo? Experiments from *C. elegans* suggest that this last possibility might occur in other systems.

## Huwe1 influences axon development

Thus far, we have detailed the extensive progress made in understanding Huwe1 function and mechanisms that impact early nervous system development. We now pivot to discuss a growing body of evidence that indicates Huwe1 has other roles later in development and in neuron function. We begin with the effects of Huwe1 on axon development.

Initial studies on cultured ES cells differentiated into neurons [15] and in cerebellum [20] suggested that *Huwe1* KO impairs neurite and parallel fiber axon growth. While this hinted at a possible role for Huwe1 in axon development, these defects could simply arise from impaired differentiation. However, findings from other systems discussed below suggest the question of whether Huwe1 affects axon outgrowth might warrant further more direct evaluation.

The first clear indication that Huwe1 can influence axon development arose from experiments in Drosophila. Flies overexpressing exogenous human HUWE1 displayed increased terminal axon branching of dorsal cluster neurons [28]. Genetic results indicated HUWE1 causes excess axon branching by negatively regulating Wnt signaling most likely via effects on Dishevelled. This is similar to results showing that mammalian Huwe1 and *C. elegans* EEL-1 inhibit Wnt signaling by effecting Dishevelled [26]. Despite this interesting finding, it is notable that we still know nothing about how *Huwe1* loss of function affects nervous system development and function in flies. This remains rich ground for future experiments, particularly given the rapid, powerful genetics of the Drosophila system.

Subsequent studies in *C. elegans* combined *eel-1* (lf) mutants with enhancer genetics to explore how EEL-1 affects axon development. The sensitizing genetic background used involved mutants for components of a highly conserved, atypical Skp/Cullin/F-box (SCF) ubiquitin ligase complex that consists of the RPM-1 E3 ubiquitin ligase and the F-box protein FSN-1 [31–34]. The RPM-1/FSN-1 ubiquitin ligase complex is an important regulator of axon termination in *C. elegans* [35, 36], and functions as both a ubiquitin ligase and a signaling hub to affect numerous downstream signaling pathways [31, 37–42]. In two different *eel-1* (lf) mutants, low levels of axon termination defects were observed in two types of neurons, the SAB motor neurons and the mechanosensory neurons that sense gentle touch [18]. Interestingly, *eel-1; fsn-1* double mutants had strong enhancer effects indicating that EEL-1 and FSN-1 function in parallel pathways to regulate axon termination. *rpm-1 (lf)* mutants have more severe, higher frequency axon termination defects than *fsn-1* mutants, because RPM-1 functions as both a ubiquitin ligase and a signaling hub. *rpm-1; eel-1* double mutants showed no increase in axon termination defects indicating that EEL-1 and RPM-1 function in the same pathway to regulate axon termination.

Mammalian cell-based experiments have shown that Huwe1 and the human RPM-1 ortholog, called Protein associated with Myc (PAM) or Myc binding protein 2 (MYCBP2), can interact in non-neuronal cell lines [43]. However, there is no evidence that this occurs in neurons or in *C. elegans*, and EEL-1 was not noted in recent comprehensive proteomic studies with RPM-1 [42]. Like HUWE1, human PAM binds MYC [44], but the domain in PAM required for this interaction is not conserved in *C. elegans* RPM-1 [31]. Moreover, axon termination defects in *eel-1* (lf) mutants were not suppressed by null alleles of *mml-1,* the *C. elegans* MYC ortholog. Thus, it seems unlikely that RPM-1 and EEL-1 function in the same pathway to regulate axon termination by affecting MYC. At present, a molecular explanation for why EEL-1 and RPM-1 function in the same pathway remains absent, but this will hopefully become more clear as proteomic and genetic studies continue with both molecules.

Overall, experiments from both flies and worms indicate that Huwe1 is a conserved regulator of axon development. However, the use of sensitizing genetic backgrounds in *C. elegans* and effects of Huwe1 on axon branching in a subset of neurons in flies suggest these are likely to be less prominent Huwe1 functions. Because Huwe1 inhibits Wnt signaling to affect neuroblast migration in worms and axon branching in flies, it is possible other genetic programs Huwe1 affects to regulate differentiation or migration might influence axon termination or branching later in development. Whether this is the case or not awaits further experiments more temporally and anatomically focused on axon development.

## Huwe1 affects inhibitory GABAergic and Glycinergic neuron function

High levels of Huwe1 expression persist in neurons broadly across the adult nervous systems of humans, mice and *C. elegans* [16–18] (Fig. 1B, C). In both mouse and human, Huwe1 expression is particularly high in superficial cortex, hippocampus and cerebellum. One possible explanation for these observations is that Huwe1 expression must be maintained in mature neurons to prevent cell proliferation. Alternatively, widespread expression might occur because Huwe1 has other roles in neuron development or function beyond its effects on neural progenitor proliferation, differentiation and migration during early nervous system development. Further support for this second possibility came from the observation that Huwe1 is present in synaptosomes isolated from mouse brain [45]. Consistent with this, imaging in live *C. elegans* found that EEL-1/Huwe1 localizes to the presynaptic terminals of inhibitory GABAergic neurons [18]. In cultured mouse spinal cord neurons, Huwe1 is present at or near postsynaptic terminals [46]. These observations indicate that in both worms and mammals Huwe1 is present at synapses where it would be capable of influencing neuron function.

**Fig. 3:**
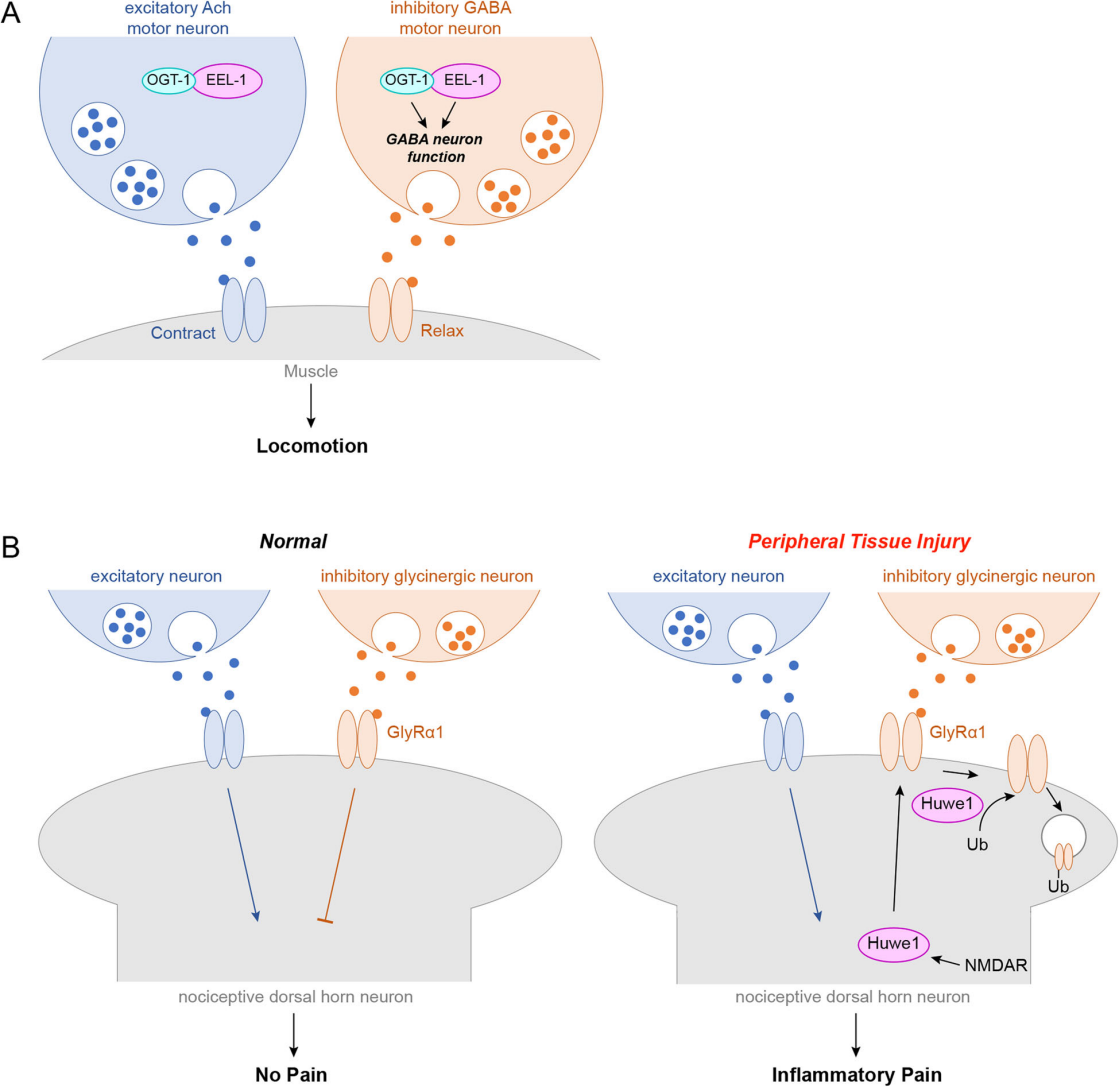
Huwe1 regulates inhibitory neurotransmission. **a** Schematic of a *C. elegans* motor circuit showing that EEL-1/HUWE1 is expressed in excitatory cholinergic and inhibitory GABAergic motor neurons, but preferentially affects GABAergic transmission. EEL-1 forms a complex with OGT-1 and functions parallel to OGT-1 to regulate GABA neuron function. **b** Schematic of excitatory and inhibitory connections to a mouse dorsal horn neuron in the spinal cord. Normally (left), glycinergic transmission inhibits excitatory input to nociceptive neurons. After peripheral tissue damage (right), Huwe1 is required to reduce glycinergic inhibitory transmission that stimulates inflammatory pain. This requires NMDAR-dependent recruitment of Huwe1, which ubiquitinates GlyRα1 and triggers GlyRα1 endocytosis (right). For A, diagram originally published in the Journal of Biological Chemistry. Giles, Desbois, Opperman, Tavora, Maroni, Grill. J. Biol. Chem. 2019; 294: 6843–6856.© Giles, Desbois, Opperman, Tavora, Maroni, Grill

The first functional evidence indicating that Huwe1 affects neurotransmission and neuronal function emerged from in vivo studies using *C. elegans* [18]. This relied upon both genetic loss- and gain-of-function approaches, pharmacological manipulation, and electrophysiological readouts using the inhibitory GABAergic motor neurons. The motor circuit of *C. elegans* controls locomotion and is composed of opposing cholinergic and GABAergic motor neurons that excite or inhibit body wall muscles, respectively (Fig. 3a). The circuit can be manipulated and studied pharmacologically using aldicarb, an acetylcholine esterase inhibitor. Aldicarb blocks acetylcholine (Ach) clearance from neuromuscular junctions leading to excess cholinergic signaling, increased muscle contraction, and eventually paralysis. There are two general principles that emerge from a large number of studies with aldicarb [47]. 1) Mutants with defective Ach function have slower build-up of Ach leading to slower paralysis, or resistance to aldicarb. 2) In contrast, mutants with impaired inhibitory GABA neuron function have reduced inhibition onto muscles. As a result, aldicarb treatment and Ach accumulation acts more quickly resulting in more rapid paralysis, or hypersensitivity to aldicarb. *eel-1* null mutants are hypersensitive to aldicarb compared to wild-type animals suggesting that EEL-1 affects GABA neuron function [18]. EEL-1 overexpression results in the opposing phenotype with slower paralysis in response to aldicarb, suggesting that GABA neuron function is increased. Electrophysiological recordings confirmed that *eel-1* mutants have robust defects in GABAergic presynaptic transmission, but normal cholinergic transmission. Genetic results also demonstrated that EEL-1 functions cell autonomously in GABA neurons to regulate presynaptic transmission, consistent with EEL-1 localization at GABAergic presynaptic terminals. Thus, EEL-1 is expressed in both cholinergic excitatory motor neurons and inhibitory GABAergic motor neurons, but predominantly influences GABAergic transmission (Fig. 3a). Importantly, effects of EEL-1 on synapse formation in GABA neurons was ruled out indicating defects in GABAergic transmission in *eel-1* mutants most likely stem from direct effects on presynaptic transmission.

There is some evidence EEL-1 has a more modest role in synapse formation [18]. Although electrophysiological results indicate synaptic transmission is not impaired in the cholinergic motor neurons of *eel-1* mutants, mild defects in synapse formation were observed in these neurons. While GABA neurons formed synapses normally in *eel-1* mutants, the use of an *fsn-1* sensitizing genetic background revealed a role for EEL-1 in synapse formation in GABA neurons. Similar to genetic outcomes with axon termination, *eel-1* and *rpm-1* function in the same pathway to regulate synapse formation in both cholinergic and GABAergic motor neurons. Thus, EEL-1 plays a prominent role in GABAergic presynaptic transmission, and has less pronounced effects on synapse formation.

Initial efforts to decipher how EEL-1 regulates GABAergic transmission relied upon in vivo affinity purification proteomics using *C. elegans* [48]. Importantly, proteomics was facilitated by the widespread expression of EEL-1 across the worm nervous system, despite the preferential effects of EEL-1 on GABAergic transmission. O-linked β-N-acetylglucosamine transferase 1 (OGT-1) was identified as a prominent EEL-1 binding protein, and this interaction was confirmed by coimmunoprecipitation from *C. elegans*. OGT-1 is a highly conserved glycosyltransferase that post-translationally modifies many cytosolic and nuclear proteins [49, 50]. OGT is broadly expressed in the nervous system of mammals including high expression in the brain, and it is localized to synapses where it modifies synaptic proteins [51–53]. In *C. elegans*, OGT-1 was localized to presynaptic terminals of GABA motor neurons which is a reasonable subcellular location for OGT-1 to bind and function with EEL-1 [48]. Results with automated aldicarb assays showed that *ogt-1* mutants were hypersensitive to aldicarb suggesting that OGT-1 regulates GABA neuron function similar to EEL-1. Analysis of *ogt-1; eel-1* double mutants, and transgenic rescue experiments showed that OGT-1 and EEL-1 function cell autonomously in parallel to regulate GABA neuron function, but do not affect synapse formation in these neurons [48]. The most likely explanation for these findings is that OGT-1 and EEL-1 work together in a complex to affect GABAergic transmission (Fig. 3a). While EEL-1 ubiquitin ligase activity is required for GABA neuron function, outcomes from genetic, proteomic and biochemical experiments suggest that OGT-1 is unlikely to be an EEL-1 ubiquitination substrate [48]. A better understanding of the relationship between EEL-1 and OGT-1, and how the EEL-1/OGT-1 protein complex affects GABA neuron function now awaits further study. Importantly, more work is encouraged by the finding that human OGT binds HUWE1 indicating that this interaction is conserved [48].

Interestingly, transgenic rescue experiments demonstrated differing roles for the catalytic activity of EEL-1 and OGT-1 in GABA neuron function. While EEL-1 ubiquitin ligase activity was required for GABA neuron function, OGT-1 glycosyltransferase activity was dispensable [48]. Thus, EEL-1 and OGT-1 likely converge on a single molecular target or cellular process that affects GABA neuron function, but this cannot simply be the result of both ubiquitination and glycosylation activity. Rather, OGT-1 is likely to have a novel molecular function as part of a complex with EEL-1. The nature of this molecular mechanism remains unknown, but could result from lesser studied roles of OGT as a scaffold protein [49, 54].

It is unknown if Huwe1 regulates GABAergic transmission in mammals, but recent work indicates that Huwe1 affects another type of inhibitory neurotransmission, glycinergic transmission [46]. Cell-based experiments with HEK 293 cells showed that Huwe1 can directly ubiquitinate Glycine receptor α1 (GlyRα1). Huwe1 is present in cultured dorsal horn neurons where it localizes to a small percentage of postsynaptic terminals. Ubiquitination of GlyRα1, which is stimulated by excitation with NMDA, is blocked when *Huwe1* is knocked down in spinal dorsal horn. The cellular effects of Huwe1 on GlyRα1 were determined by examining endocytosis in cultured spinal neurons [46]. *Huwe1* knockdown reduced activity-induced surface labeling and internalization of GlyRα1 indicating that Huwe1 is required for GlyRα1 endocytosis.

To address the functional effects of *Huwe1* on glycinergic transmission, *Huwe1* was knocked down and electrophysiology was performed on spinal cord slice. *Huwe1* knockdown resulted in failure to decrease inhibitory glycinergic transmission following an inflammatory challenge. Consistent with this, *Huwe1* knockdown blunted pain responses to inflammation. It is notable that *Huwe1* knockdown did not affect baseline glycinergic transmission in the absence of an inflammatory challenge. This could be because Huwe1 only modifies glycinergic transmission in the context of inflammation, or because *Huwe1* knockdown does not completely abolish Huwe1 function. These results demonstrate that after tissue injury Huwe1 ubiquitinates GlyRα1 to decrease inhibitory glycinergic neurotransmission and facilitate pain sensation (Fig. 3b). Experiments with *Huwe1* null animals will be important in further assessing Huwe1 effects on glycinergic transmission in the spinal cord. Nonetheless, existing findings suggest that Huwe1 could be an important new molecular target for treating inflammatory pain.

Thus, results from *C. elegans* and rodent model systems indicate that Huwe1 has a conserved role in regulating inhibitory neurotransmission. While much remains unknown about how EEL-1 regulates GABAergic transmission, initial outcomes from in vivo proteomics and genetics indicates that EEL-1 forms a complex with OGT-1, and functions parallel to OGT-1 to regulate GABA neuron function. Continued exploration of the proteomic and genetic space surrounding EEL-1 using invertebrate models like *C. elegans* is ideally positioned to further reveal how EEL-1 regulates GABAergic transmission. The discovery that Huwe1 regulates inhibitory glycinergic neurotransmission in spinal cord following tissue injury is another prominent step forward. These findings across systems prompt an important question: Does Huwe1 regulate GABAergic and/or glycinergic inhibitory neurotransmission in the brain?

## Huwe1 in mitochondrial function and degradation

We now turn to emerging evidence that Huwe1 can effect mitochondria. This includes regulation of mitochondrial function and fragmentation, as well as mitochondrial turnover via mitophagy. Huwe1 effects on mitochondria could have important implications in the nervous system where mitochondria provide energy and calcium buffering for neuron function, and altered turnover of mitochondria is implicated in nervous system disease.

Mitofusins are key regulators of mitochondrial fusion and function, affect neurodegeneration, and are causally implicated in Charcot-Marie-Tooth (CMT) Disease which features peripheral axon degeneration [55–58]. Huwe1 regulates Mitofusin 2 (Mfn2), the Mitofusin isoform that is involved in CMT and neurodegeneration [59]. This discovery stemmed from affinity purification proteomics that identified Huwe1 as an Mfn2 binding protein in cell lines. Cell-based experiments using HEK 239 cells showed this interaction involves the BH3 domain of Huwe1, and is dependent upon serine phosphorylation of Mfn2. *Huwe1* knockdown prevented degradation of Mfn2 and mitochondrial fragmentation in response to pharmacologically-induced stress in the U2OS non-neuronal cell line. Consistent with Huwe1 ubiquitinating and inhibiting Mfn2, *Huwe1* knockdown results in increased U2OS cell death, which is suppressed by simultaneous *Mfn2* knockdown [59]. A subsequent study using S2 insect cells showed that steric acid signaling opposes Huwe1 effects on Mfn2 thereby influencing mitochondrial fragmentation [60].

Huwe1 can also affect mitophagy, a specific form of autophagy that allows removal of damaged mitochondria. Mitophagy is regulated by the pro-autophagy molecule autophagy/beclin-1 regulator-1 (AMBRA1). Affinity purification proteomics using Hela cells stimulated to undergo mitophagy identified Huwe1 as a an AMBRA1 binding protein [61]. Mitophagy induced by mitochondrial membrane targeting of AMBRA1 was rescued by *Huwe1* knockdown. This result suggests that AMBRA1 functions through Huwe1 to influence mitophagy. This is further supported by the observation that Huwe1 ubiquitination of Mfn2 was increased by coexpression of membrane targeted AMBRA1. These results indicate that AMBRA1 functions with Huwe1 to regulate Mfn2 degradation and mitophagy. Interestingly, Huwe1 also ubiquitinates AMBRA1 leading to AMBRA1 phosphorylation and induction of mitophagy. This suggests that Mfn2 and mitophagy are affected by a complex signaling relationship between Huwe1 and AMBRA1.

While these are important discoveries, effects of Huwe1 on mitochondria and mitophagy have only been shown in non-neuronal cell lines to date. Whether Huwe1 degrades Mfn2 to affect mitochondrial function, fragmentation and mitophagy in the nervous system remains unknown. However, if this is the case, it would have intriguing implications for nervous system health and disease.

## *HUWE1* copy number variations and non-syndromic intellectual disability

The first genetic links between human *HUWE1* and neurodevelopmental disorders emerged with the discovery that X-chromosome microduplications that lead to copy number variations (CNV) in *HUWE1* are associated with non-syndromic, X-linked ID [62–64]. One of these studies also identified missense mutations in *HUWE1* associated with non-syndromic X-linked ID [64], which we discuss later. Since these early discoveries, a plethora of human genetic studies have linked *HUWE1* genetic changes with ID. While genetic changes in *HUWE1* are associated with other neurodevelopmental conditions, such as seizure/epilepsy, autism and schizophrenia, we primarily focus on ID because it has the most compelling links to *HUWE1*. Patients with *HUWE1* genetic changes do have developmental abnormalities outside of the nervous system (for example slowed growth and altered facial features) that we do not comment on here. We believe a discussion of these topics is better left to a review with a greater clinical focus. Nonetheless, we hope our review encourages further studies and commentary aimed at determining whether particular genetic changes in *HUWE1* represent one or many molecularly-defined syndromes. This already appears to be well under way in one case, that of Juberg-Marsidi-Brooks syndrome [65].

Microduplications of chromosome Xp11.22 which result in CNV in *HUWE1* were initially identified independently by two groups using microarray-comparative hybridization approaches [63, 64] (Table 1). These two studies showed that *HUWE1* is a common gene present in all Xp11.22 microduplications identified from 7 families. Moreover, in one study microduplications containing *HUWE1* were shown to segregate with ID in 6 of these families and were not identified in control samples [64]. Importantly, this study also performed qPCR and showed that *HUWE1* expression levels were elevated in these patient samples, consistent with an overexpression/gain-of-function effect on HUWE1. Subsequent studies that used exome or next generation sequencing identified *HUWE1* CNVs in numerous other families with non-syndromic ID [66–75] (Table 1).

**Table 1.**
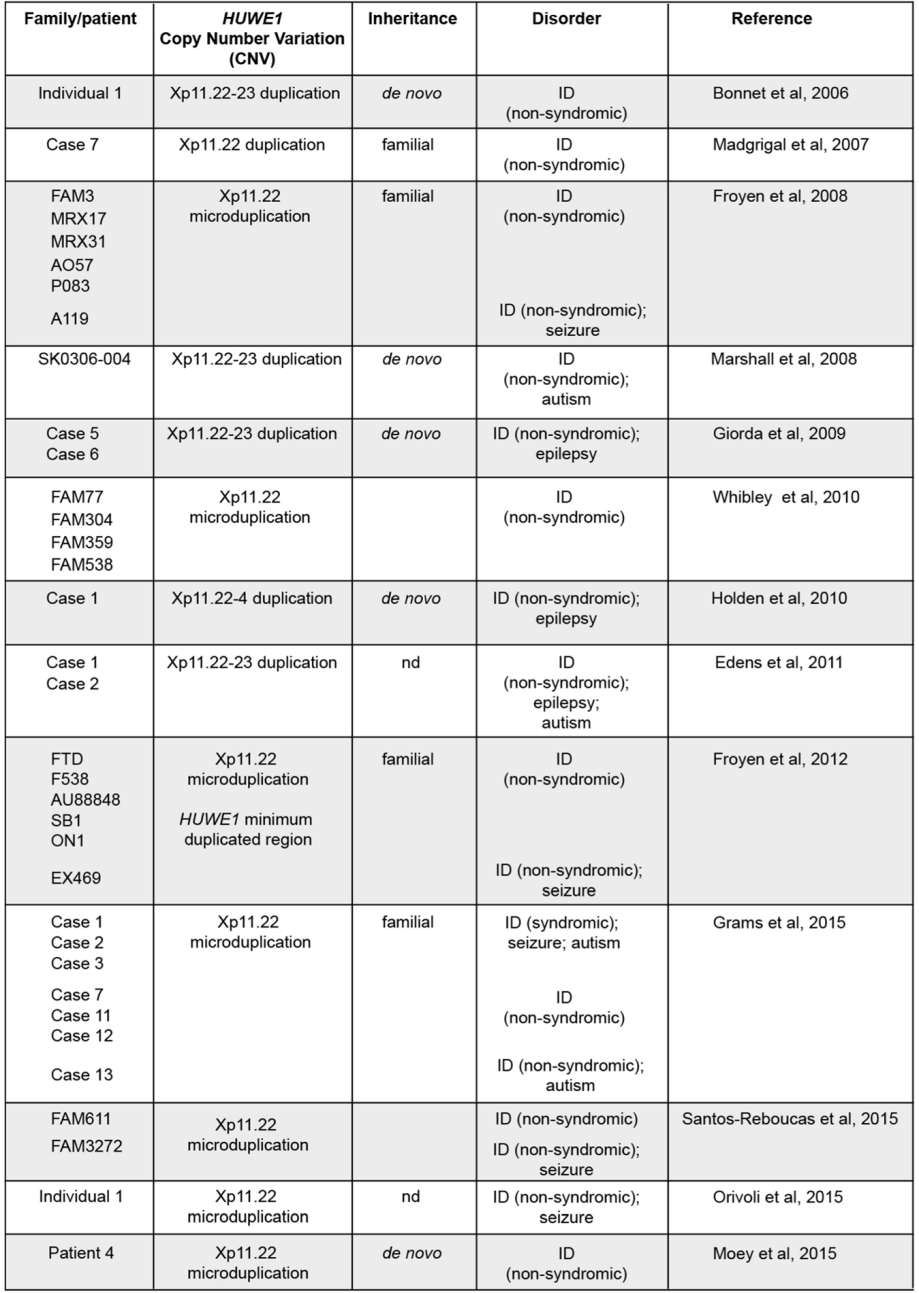
*HUWE1* copy number variations associated with non-syndromic ID.

One particular study was critical in establishing the link between *HUWE1* CNV and ID [71]. Froyen and colleagues identified 6 families with Xp11.22 microduplications containing *HUWE1*, showed that *HUWE1* was the only common gene duplicated, and demonstrated that HUWE1 expression was increased in individuals with ID. Importantly, one of these Xp11.22 microduplications did not include *HSD17B10*, the gene adjacent to *HUWE1*. Similar findings ruled out two microRNAs, *miRNA98* and *let7f-2*, that are also often present with *HUWE1* in Xp11.22 microduplications. Through a compelling collective effort, the field now has data from 35 different families/individuals that convincingly argues increased HUWE1 function is likely to cause non-syndromic ID (Table 1).

Individuals with *HUWE1* CNVs sometimes have other neurodevelopmental conditions that accompany ID. The most compelling links are to epilepsy or seizure [64, 67, 69–74]. Autism-like behaviors have also been documented [66, 70, 72]. While seizure/epilepsy and autism are observed in some individuals with HUWE1 CNVs, the genetic links between HUWE1 and these neurodevelopmental conditions are much less clear and less consistent than ID. Thus, increased HUWE1 function might contribute to these outcomes, but it is also possible that they are caused by other genes present in duplications that contain *HUWE1*.

The concept that increased HUWE1 function leads to ID is further supported by outcomes from both vertebrate and invertebrate animal models, which show that increased HUWE1 function is detrimental to the nervous system. For example, overexpression of HUWE1 in cortical neurons results in reduced proliferation of neural progenitors [19]. In *Drosophila*, overexpression of HUWE1 results in altered axon branching in some types of neurons [28]. Finally, work in *C. elegans* showed that overexpressing HUWE1 in GABAergic neurons alters their function [18]. Thus, findings from three different in vivo model systems indicate that increased HUWE1 function can adversely affect neuron function and development. Despite this progress, we still know relatively little about how increasing HUWE1 function affects different brain regions and different types of neurons in vivo. Moreover, which HUWE1 signaling mechanisms are the most important to phenotypes caused by HUWE1 overexpression remains entirely unclear.

## *HUWE1* mutations in syndromic and non-syndromic intellectual disability

**Fig. 4:**
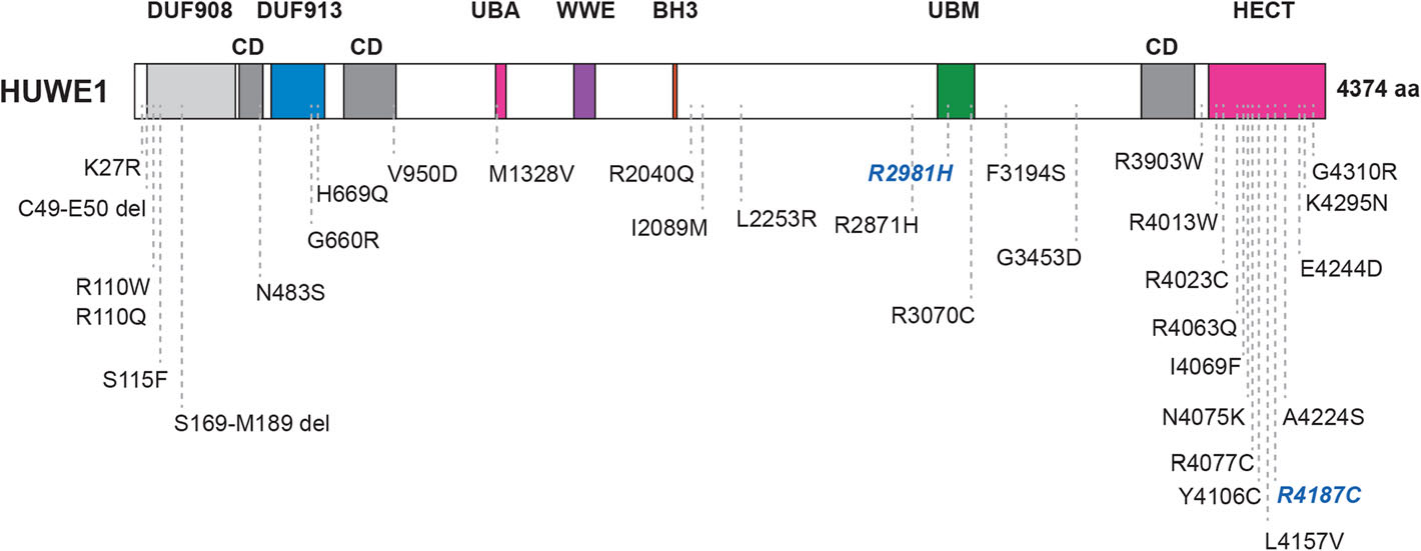
Mutations in HUWE1 associated with non-syndromic and syndromic forms of X-linked Intellectual Disability. Schematic of human HUWE1 and amino acid changes resulting from ID-associated mutations in *HUWE1*. ID-associated mutations occur broadly across the HUWE1 protein sequence with two hotspots, the HECT domain and DUF908 domain. Blue italics highlight two ID-associated mutations that were shown to result in loss-of-function when tested using in vivo assays of neuron function in *C. elegans*. Note three Conserved Domains (CD) of unknown function are annotated

A growing number of human genetic studies have also identified *HUWE1* missense mutations that are associated with both syndromic and non-syndromic forms of X-linked ID. The first *HUWE1* mutations identified in three families with non-syndromic ID were R4013W, R2981H and R4187C [64, 76] (Table 2, Fig. 4). All three arginine residues (and motifs containing them) are highly conserved from *C. elegans* through humans, and two residues are in the HECT ubiquitin ligase domain. Experiments using *C. elegans* showed two of these exact disorder-associated mutations engineered into EEL-1/HUWE1 fail to rescue GABA neuron defects in *eel-1* (lf) mutants [18] (Fig. 4, blue italics). In contrast, wild-type EEL-1 does rescue *eel-1* (lf) defects. Thus, results from in vivo experiments demonstrate that two ID-associated mutations impair EEL-1 function, and therefore are likely to impair HUWE1 as well. We note that these point mutations are probably not *HUWE1* null alleles for two reasons. 1) *Huwe1* knockout mice display neonatal lethality [19, 20]. 2) More damaging large deletion alleles have not been identified for *HUWE1* despite large numbers of human genetic studies [71].

**Table 2.**
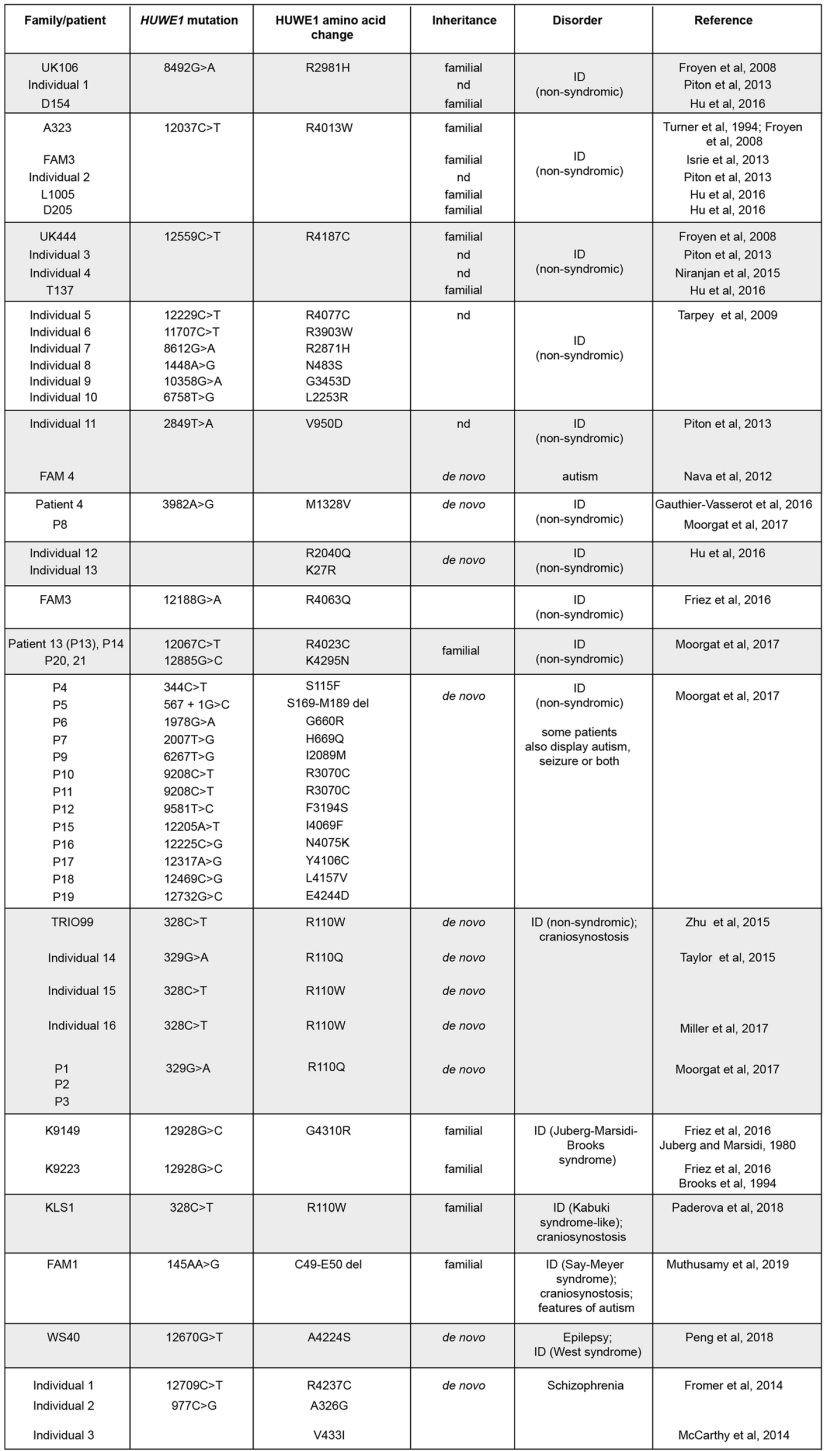
*HUWE1* missense mutations associated with neurodevelopmental disorders including non-syndromic and syndromic ID

Independent studies identified numerous other missense mutations in *HUWE1* associated with non-syndromic ID that show familial inheritance or occur de novo in individuals [65, 77–83] (Table 2; Fig. 4). To date, a total of 31 genetic changes in *HUWE1* isolated from 49 different families or individuals with non-syndromic ID have been identified (Table 2; Fig. 4). Importantly, several mutations (R4013W, R2981H, R4187C, R110Q/W and M1328V) were identified in multiple different families/individuals (Table 2). This high quality, extensive progress with human genetics makes a strong case that mutations in *HUWE1* are likely to cause non-syndromic ID.

There are other important points to be made based on the impressive scope of these studies. First, mutations in *HUWE1* associated with non-syndromic ID cause amino acid changes that are broadly distributed across the entire HUWE1 protein (Fig. 4). Mutations affect annotated domains of unknown function (DUF 908, DUF 913), unannotated evolutionarily conserved domains (CD) of unknown function, the UBA domain, the UBM domain, and the HECT catalytic ubiquitin ligase domain (Fig. 4). With that said, there are two domains in HUWE1 that are the most frequently mutated, and possible ID hot spots. These are the N-terminal DUF 908 domain (5 mutations) and, even more so, the C-terminal HECT domain (13 mutations) (Fig. 4). These findings suggest that HUWE1 ubiquitin ligase activity is likely to be involved in ID, and other domains harboring ID-associated mutations are likely to be important for HUWE1 function or structure.

Comparison of ID-associated mutations with the HECT domain crystal structure revealed further valuable insight. Two mutations, E4244D and K4295N, affect residues required for HECT domain structure [13] (Fig. 4). Another mutation, R4013W, is adjacent to K4014, another structurally important residue [13]. The L4157V mutation affects a residue that is required for binding to the E2 enzyme, suggesting it could affect substrate ubiquitination [13]. Mutations occur in two domains that bind ubiquitin [84, 85], the UBA (M1328V) and UBM (R2981H, R3070C) domains (Fig. 4). While the structural and functional roles of the UBA and UBM domains in HUWE1 remain unclear, it is possible mutations in these domains might affect binding to ubiquitin thereby influencing the efficiency of substrate ubiquitination or the stability of ubiquitin modifications on substrates. No mutations were found in a small region (D3951 to L3992) that mediates dimerization and inhibition of the HUWE1 HECT domain [14] (Fig. 4). Collectively, these observations suggest that ID-associated mutations are likely to impair HUWE1 function.

Other neurodevelopmental conditions can show comorbidity with non-syndromic ID in some individuals with *HUWE1* mutations. For example, a small number of studies focused on craniosynostosis identified mutations in *HUWE1*, and also observed non-syndromic ID in these individuals (Table 2) [86–88]. In all cases, R110Q/W mutations occurred, which suggests that this residue might be particularly relevant in craniosynostosis. Alternatively, because most studies focused on non-syndromic ID might not have evaluated craniosynostosis, this could be a core, undiagnosed feature of disease associated with mutations in *HUWE1*. Indeed, one study examining non-syndromic ID identified several individuals with R110Q mutations and noted that they have craniosynostosis (Table 2) [83]. This study also observed seizures and autism as comorbidities with non-syndromic ID in some individuals with *HUWE1* mutations (Table 2) [83].

Importantly, *HUWE1* mutations have also emerged in syndromic forms of ID. Friez and colleagues used next generation exome sequencing to revisit the genetic basis of two syndromic forms of ID, Juberg-Marsidi and Brooks syndromes [65] (Table 2). Identical *HUWE1* mutations, G4310R, were identified in patients from both of these syndromes. This led the authors to reclassify this as a single syndromic form of ID, Juberg-Marsidi-Brooks (JMB) syndrome, that has the same molecular genetic basis. Cell-based experiments using cells derived from these individuals showed that HUWE1 ubiquitination substrates were increased [65]. Thus, the G4310R lesion is likely to result in loss of *HUWE1* function, but is likely to be a hypomorphic allele for reasons discussed above. Another possible link between mutations in *HUWE1* and syndromic ID involves a patient with a Kabuki-like syndrome (Table 2) [89]. Interestingly, this individual has the same mutation, R110W, that was identified in several individuals with craniosynostosis and non-syndromic ID (Table 2) [86–88]. Finally, a splicing mutation that deletes two amino acids from HUWE1 was found in a family that potentially has Say-Meyer syndrome, another syndromic form of ID (Table 2) [90]. Taken as a whole, these studies suggest that mutations in *HUWE1* potentially result in several types of syndromic ID, including a potential causal link to JMB. While this is important progress, identification of *HUWE1* mutations in more families with JMB, and other syndromic forms of ID, would be valuable.

*HUWE1* missense mutations can be associated with other neurodevelopmental conditions. For example, a variant in *HUWE1* was found in an individual with West syndrome, a form of early infantile epileptic encephalopathy that has accompanying ID (Table 2) [91]. De novo mutations in *HUWE1* were found in individuals with schizophrenia [92, 93] and possibly autism [94] (Table 2). Notably, this single patient with autism has the same mutation, V950D, that was identified in a different individual with non-syndromic ID (Table 2) [79]. Thus, ID and autism could be comorbidities associated with mutations in *HUWE1*, or certain *HUWE1* mutations could be risk factors for these conditions such that ID and autism can occur separately or together. Answers to these questions now await further more extensive behavioral and clinical studies on individuals with *HUWE1* mutations.

Further links between HUWE1 and ID have emerged from identification of proteins that interact with HUWE1, and studies that evaluated *HUWE1* RNA expression. Proteomics identified the glycosyltransferase OGT as a conserved binding protein for both *C. elegans* EEL-1/HUWE1 and human HUWE1 [48]. OGT was found to function with EEL-1 to regulate inhibitory GABA neuron function. Similar to *HUWE1*, human genetic studies have identified *OGT* mutations in multiple families with non-syndromic ID [80, 95–98]. To date, 7 different mutations in *OGT* have been found in 11 individuals with ID. Familial inheritance indicates that *OGT* mutations segregate with ID, providing further support for the involvement of OGT in ID [96–98].

There is one last intriguing link between HUWE1 and ID. Findings from mice and Drosophila showed that *HUWE1* mRNA is a prominent, conserved target of the RNA binding protein FMRP, and FMRP affects *HUWE1* RNA levels [99, 100]. This is a potentially important observation as mutations in *FMRP* cause Fragile X syndrome, a highly prevalent syndromic form of ID. Whether *HUWE1* is a relevant target of FMRP in regard to pathology, behavioral abnormalities or cognitive impairment in Fragile X syndrome remains unclear, but is certainly an interesting possibility given mounting evidence linking *HUWE1* genetic changes to ID.

In summary, a compelling body of human genetic studies and work using both mammalian and invertebrate model systems support several overarching concepts regarding *HUWE1* and neurodevelopmental disorders. 1) There is extensive evidence that both CNVs and mutations in *HUWE1* likely cause non-syndromic ID. Thus, both gain- and loss-of-function genetic changes in *HUWE1* are likely to result in ID. This is similar to other prominent players in ID, such as *MECP2* and *SHANK3* [101]. 2) Mutations in *HUWE1* are potentially associated with several syndromic forms of ID with the most substantive links to JMB syndrome. 3) Further evidence linking HUWE1 to ID has emerged from protein interaction and RNA expression studies. This suggests that HUWE1 could be part of signaling networks involved in ID.

## Conclusion

Over the past decade, HUWE1 has emerged as a relevant player in several neurodevelopmental disorders with the most prominent links to ID. As a result, several future research directions have become particularly compelling. It will be imperative that we comprehensively unravel the mechanisms by which HUWE1 regulates nervous system function and development in order to identify potential therapeutic targets and strategies for molecular intervention. In vivo invertebrate and vertebrate models are likely to be invaluable in this process. It is critical to further investigate how precise *HUWE1* genetic changes that occur in ID affect neuron function and development. Finally, we hope that continued human genetic and clinical studies will allow for more comprehensive, precise molecular genetic classification of *HUWE1* neurodevelopmental disorders. While identification of the molecular genetic basis of JMB is a valuable start, much important work remains to be done.

## Data Availability

Not applicable.
